# An Adaptive Learning Model for Multiscale Texture Features in Polyp Classification via Computed Tomographic Colonography

**DOI:** 10.3390/s22030907

**Published:** 2022-01-25

**Authors:** Weiguo Cao, Marc J. Pomeroy, Shu Zhang, Jiaxing Tan, Zhengrong Liang, Yongfeng Gao, Almas F. Abbasi, Perry J. Pickhardt

**Affiliations:** 1 Department of Radiology, Stony Brook University, Stony Brook, NY 11794, USA; Weiguo.Cao@stonybrookmedicine.edu (W.C.); marc.pomeroy@stonybrook.edu (M.J.P.); shuzhang8967@163.com (S.Z.); Yongfeng.Gao@stonybrookmedicine.edu (Y.G.); almas.abbasi@stonybrookmedicine.edu (A.F.A.); 2 Department of Biomedical Engineering, Stony Brook University, Stony Brook, NY 11794, USA; 3 Department of Computer Science, City University of New York, New York, NY 10314, USA; jtan@gradcenter.cuny.edu; 4 Department of Radiology, School of Medicine, University of Wisconsin, Madison, WI 53792, USA; PPickhardt2@uwhealth.org

**Keywords:** colorectal cancer, computed tomographic colonography, polyp classification, texture features, random forest, convolutional neural network

## Abstract

**Objective:** As an effective lesion heterogeneity depiction, texture information extracted from computed tomography has become increasingly important in polyp classification. However, variation and redundancy among multiple texture descriptors render a challenging task of integrating them into a general characterization. Considering these two problems, this work proposes an adaptive learning model to integrate multi-scale texture features. **Methods:** To mitigate feature variation, the whole feature set is geometrically split into several independent subsets that are ranked by a learning evaluation measure after preliminary classifications. To reduce feature redundancy, a bottom-up hierarchical learning framework is proposed to ensure monotonic increase of classification performance while integrating these ranked sets selectively. Two types of classifiers, traditional (random forest + support vector machine)- and convolutional neural network (CNN)-based, are employed to perform the polyp classification under the proposed framework with extended Haralick measures and gray-level co-occurrence matrix (GLCM) as inputs, respectively. Experimental results are based on a retrospective dataset of 63 polyp masses (defined as greater than 3 cm in largest diameter), including 32 adenocarcinomas and 31 benign adenomas, from adult patients undergoing first-time computed tomography colonography and who had corresponding histopathology of the detected masses. **Results:** We evaluate the performance of the proposed models by the area under the curve (AUC) of the receiver operating characteristic curve. The proposed models show encouraging performances of an AUC score of 0.925 with the traditional classification method and an AUC score of 0.902 with CNN. The proposed adaptive learning framework significantly outperforms nine well-established classification methods, including six traditional methods and three deep learning ones with a large margin. **Conclusions:** The proposed adaptive learning model can combat the challenges of feature variation through a multiscale grouping of feature inputs, and the feature redundancy through a hierarchal sorting of these feature groups. The improved classification performance against comparative models demonstrated the feasibility and utility of this adaptive learning procedure for feature integration.

## 1. Introduction

Colorectal cancer (CRC) is one of the top fatal diseases in the United States. American Cancer Society ranks CRC as the third most common cancer and the third leading cause of cancer-related deaths in both men and women [1]. Because most colon cancers are developed from precursor polyps, polyp screening has become the primary means for CRC prevention [2,3]. Computed tomographic colonography (CTC), as a minimally invasive polyp detection tool, has become an important alternative method in polyp screening and management [4]. This CTC technology has shown its potential in physicians’ hands with computer-aided detection tools to localize polyps in practice [5]. For personalized precision medicine, it is very important to know the pathological subtype of (or to diagnose) the detected polyps for optimal treatment. Yet, diagnosis from the CTC images with subtle image contrast within the polyp volume is very challenging in the radiologist experts’ hands with current existing computer aided diagnosis (CADx) tools [6]. Therefore, more advanced CADx tools are needed.

Heterogeneity is a key factor in determining the malignancy of a lesion and its response to intervention [7,8]; CADx models have, therefore, focused on using image contrast patterns to quantify and describe that heterogeneity [9,10,11]. Toward that purpose, many texture patterns and descriptors have been proposed to extract and quantify texture information for CADx of polyps, such as gray level co-occurrence matrix (GLCM) [10], local binary pattern (LBP) [12,13,14], Gabor filter [15,16], wavelets [17,18], and Weber local descriptor (WLD) [14,19]. With the growth of the number of texture descriptors, how to merge them has become an important issue because of the variation in computing these texture descriptors, as well as possible redundant information among them [20,21,22].

The variation problem can occur from the method used to compute the texture patterns or to extract the texture features. For example, the traditional Haralick texture features [10] were computed by the average and range across the 13 neighbor directions through the volumetric voxel array that did not consider geometrical scale variations (or multi-scale nature) among those neighbor directions [11]. The redundancy problem has been under investigation for many years as a feature selection or dimension reduction task. This is a typical NP (non-deterministic polynomial time) hard problem [22] that has generally been approached by three classes of methods: filter methods [23,24], wrapper methods [25,26], and embedded methods [27,28,29,30,31,32,33]. Despite these advancements, how to obtain the best feature subset from all the extracted patterns and features still remains a great challenge [34,35,36,37].

To address the above, the problems of (1) variation in polyp texture descriptor computation and (2) redundancy in multiple computed patterns and features, this work proposes an adaptive fusion model for the polyp classification task. This work uses the GLCM and its measures to demonstrate this adaptive fusion model, where the second-order nature of the GLCM allows for inherent grouping by spatial distance of image voxel pairs. Two different models, using traditional classifiers and a deep learning architecture, are proposed to evaluate this adaptive fusion methodology, where input features are grouped by spatial displacement and weighted by initial classification performance. By adaptively combining these groups in order of their initial weights, the proposed models can select the most important features from the inputs, thereby reducing variation and redundancy in the final model. Both models are evaluated on a dataset of colorectal polyp masses and show significant improvement in classification performance compared to state-of-the-art methods.

## 2. Materials and Methods

This section begins with a review of the GLCM texture descriptor calculation. Then the multiscale analysis for fusing the calculated texture descriptor sets is discussed. Thereafter, the adaptive learning model is presented and analyzed.

### 2.1. Multiscale Sampling of GLCMs for Multiscale Features

**Figure 1:**
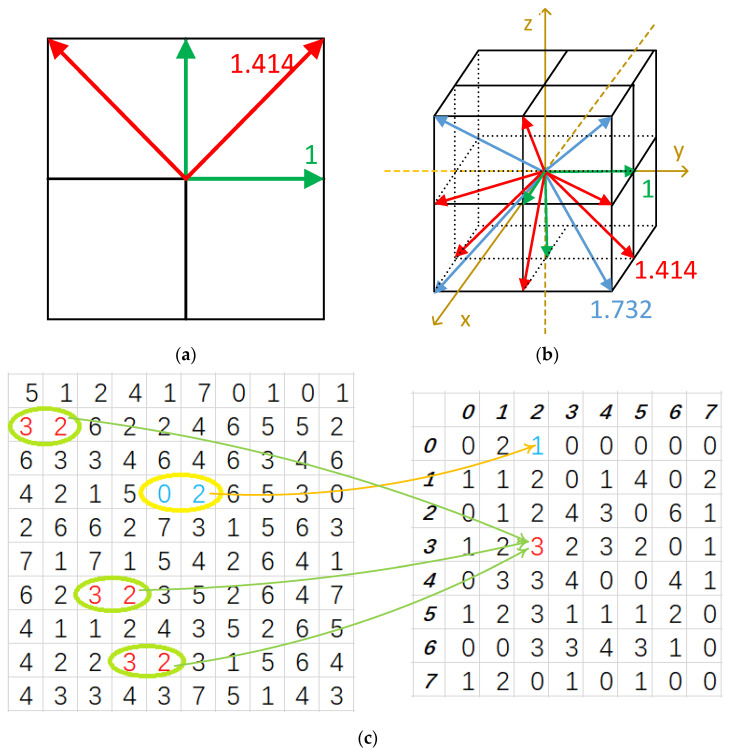
Illustration of co-occurrence matrix (CM) calculation in 2D/3D images: (**a**) CM parameters in 2D images; (**b**) CM parameters in 3D images; and (**c**) A GLCM example of a 2D case when direction is 0° and displacement = 1. The left is a gray image, and the right one is its GLCM.

Gray level co-occurrence matrix or GLCM as a typical texture pattern descriptor is widely used in medical imaging [9,10,11]. Its computation could be referred to according to the following expression in two-dimensional (2D) representation:

(1) $$C_{i,j}\;\left(d,\theta \right)=\sum_{m=1}^{M}\sum_{n=1}^{N}\{\begin{array}{cc} 1 & I\left(m,n\right)=i\;\;\&\;\;I\left(\left(m,n\right)+d\left(\theta \right)\right)=j\;\\ 0 & otherwise \end{array}$$

where *I* is the gray level image, (*M*,*N*) is the image size, indices *i* and *j* represent a pair of image pixel values, and *d(θ)* is a shifting vector between two concerned points along the direction θ, such that d(θ)=d∗(cosθ,sinθ). For 3D volumetric image data, the definition of GLCM is very similar, except that there are two angular variables in contrast to one in the 2D model. Their calculations are shown in Figure 1.

In a digital image array, the first- and second-order neighbors, which comprise the first ring around the center image voxel, are most frequently used for vector calculation. A voxel in 3D volumetric data generally has 26 neighbors, which could produce 26 vectors, including 13 vectors and 13 negative vectors. From Equation (1), it is easy to prove that the GLCM of one vector is equal to the transposed GLCM of its negative vector. Therefore, only 13 directions are preserved, while their negative vectors are all neglected in GLCM calculation due to redundant information, as shown in Figure 1b. Moreover, only the 1st ring neighbor around one concerned voxel is used; the gray level is set to be 32 in the calculation.

Many statistical measures have been proposed to quantify each GLCM for texture features. Haralick et al. proposed 14 measures, which are called Haralick measures (HMs) [10]. Hu et al. then added 16 new measures based on HM, which are donated by extended Haralick measures (eHMs) [11]. In this article, only 28 of the 30 measures from eHM are used to construct the texture descriptors (two of the 30 were proved to have limited new information and are ignored [38]) and are generated using in-house software. Therefore, the GLCM-descriptor contains 364 variables from 28 HMs over 13 directions, expressed by:

(2) $$D=\left(d_{1},\ldots \;,\;d_{364}\right)\;$$

Geometrically, the distance between the cubic center (of the first- and second-order voxel array) and the center of one neighbor voxel is not a constant and varies between 1 and $\sqrt{3}$ in terms of the voxel side unit. For example, d(. )=1 for the directions along x, y and z axes, $d\left(.\right)=\sqrt{2}$ for the diagonal directions in the 2D planes of the 3D x-y-z array coordinates, and $d\left(.\right)=\sqrt{3}$ for the diagonal directions in the 3D x-y-z array coordinates. In other words, in the discrete volumetric data, twenty-six neighbors around one voxel could produce three distances of 1, $\sqrt{2}$, and $\sqrt{3}$, i.e., a multi-scale data sampling nature. The 13 directions used to compute the GLCMs could be divided into 3 subgroups, i.e., $D_{1}$, $D_{2}$, and $D_{3}$, according to their geometric distances. Each direction within the subgroup, therefore, shares the same geometric sampling distance. Figure 1b gives the geometric interpretation. $G_{1}$ (green) contains three directions, $G_{2}$ (red) contains six directions, and, lastly, $G_{3}$ (blue) contains four directions from this subdivision. The three GLCM groups would produce three descriptors, where their corresponding variable numbers are 84 (28*3 eHMs from $G_{1}$), 168 (28*6 eHMs from $G_{2}$), and 112 (28*4 eHMs from $G_{3}$). In this manuscript, the groups of GLCMs will be given the notation $G_{i}$, and the groups of texture descriptors given the notation $D_{i}$. These descriptors could further be written by:

(3) $$\{\begin{array}{c} D_{1}=\left(\begin{array}{ccc} d_{1}^{1}, & \cdots & ,\;d_{84}^{1} \end{array}\right)\\ D_{2}=\left(\begin{array}{ccc} d_{1}^{2}, & \cdots & ,d_{168}^{2} \end{array}\right)\\ D_{3}=\left(\begin{array}{ccc} d_{1}^{3}, & \cdots & ,d_{112}^{3} \end{array}\right) \end{array}$$

The traditional Haralick texture feature calculation considered these three direction groups as one scale by computing the average and range across all 13 directions for each of the 14 traditional HMs, resulting in a total of 28 traditional Haralick texture features (HFs). For the 28 eHMs, the average and range across all 13 directions result in a total of 56 extended HFs, called eHFs. These Haralick texture features will be used as the baseline reference in this work to show the gain by the consideration of the multi-scale data sampling nature in the following. The GLCMs are then calculated by three different scales, i.e., 1, $\sqrt{2}$ ≈ 1.414 and $\sqrt{3}$ ≈ 1.732, as shown in Figure 1b. Essentially, this multi-scaling feature extraction operation is not only a direction subgrouping but also a feature subdivision. Therefore, this method generates three GLCM subgroups and three texture descriptor subdivisions, each with a different scale, as shown in Table 1. In the following, the variables in each direction group are labeled as a set of data sampled from the polyp object and treat all three direction group datasets as three differently sampled data from the same subject. Then, an adaptive machine learning strategy is developed to integrate these different datasets together for improved CADx performance by circumventing the two problems of (1) variation in polyp texture descriptor computation and (2) redundancy in multi-scale computed features.

### 2.2. Analyze Group-Specific Information

To analyze and compare the differences among the three data subsets or multi-scale groups, the information provided by each group is then investigated. To understand these differences, the information that can be learnt by CNN on each individual group is first visually analyzed. Next, CNN models based on three GLCM subgroups are trained. Then, features learnt by CNN are understood via interpreting how the final decision is made given an input.

**Figure 2:**
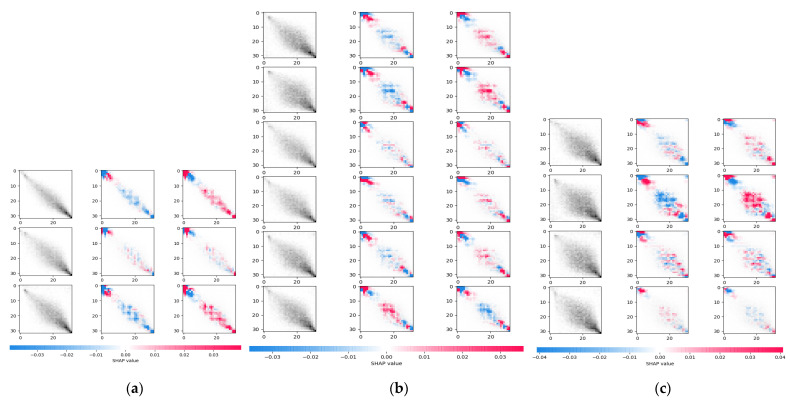
Visualization of information CNN learnt from each subgroups: (**a**) $G_{1}$, (**b**) $G_{2}$ and (**c**) $G_{3}$. The first column is the original GLCM. The corresponding label (0 for benign and 1 for malignant) and model score of the malignancy risk are listed on the top. The remaining two columns are the interpretations of model prediction on the two classes. The red cells show the entries push the model’s decision close to that class, while blue pixels pull the prediction results away.

To accomplish this, a game theory based model called SHAP was adapted to explain the output of the machine learning models [39]. Each model was trained by the polyps’ corresponding GLCM subgroup and is similar to GLCM-CNN, with network design optimized to the subgroups [40]. After the CNN model was trained, the decision criteria was visualized on the testing dataset using SHAP. Figure 2 demonstrates the learnt feature from the three subgroups by explaining the decision result of one representative polyp. The first column is the original GLCM. The corresponding label (0 for benign and 1 for malignant) and model score of the malignancy risk are listed on the top. The remaining two columns show the interpretation of model prediction on the two classes. Given a class, the red cells showed that the entries pushed the model’s decision close to that class, while blue pixels pulled the prediction results away. Based on this visualization, it can be observed that the information provided by the three subgroups had both shared patterns and unique patterns. The visualization results of these patterns from deep learning showed the potential for the proposed adaptive learning model to learn these group specific and groupwise shared features.

### 2.3. Adaptive Learning Model for Fusing Multi-Scale Features

As the variable number grows, simply combining all the input variables for classification can increase a high risk of clustering degradation, which is caused by counteractions of their variations [20,22]. In practice, not all variables of the descriptor will be useful for classification; lots of redundant information remains in the three scales. Inspired by [38], an adaptive learning model is designed to hierarchically circumvent the variation and reduce the redundant information from the multi-scale feature sets.

**Problem Formulation:** The problem is formulated as follows: Given a set $S=\{D_{i}\;|\;i\;\epsilon \;\left[1,\;n\right]\}$ containing n feature groups $D_{i}$, the task is to find an optimal set $\hat{S}\;\subset S$ that maximizes the polyp classification performance in terms of AUC. Actually, this is a famous problem of the curse of dimensionality, which is always NP-hard [41]. To avoid this problem, the greedy algorithm as the suboptimal scheme is introduced.

**Figure 3:**
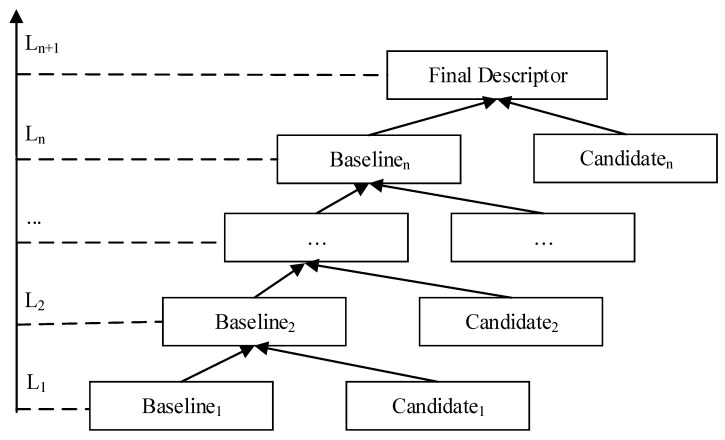
The flowchart of multi-level learning model for fusion of multi-scale feature sets.

As shown in Figure 3, the proposed adaptive learning method works in two stages: baseline selection and hierarchical feature integration. The goal of the baseline is to select the best individual group that achieves the highest performance. After ranking the rest feature groups in a descending order based on its individual performance, the multi-level integration method integrates new group one by one following the forward step feature selection (FSFS) method. Given a new feature group $D_{j}$, FSFS is designed to add new variables from the most significant to the least and to only keep the ones that have performance improvement.

Two models for the adaptive learning method are proposed. The first one is a traditional hybrid method; the second is a deep learning-based method. They are detailed below.

**Multigroup hybrid Method:** The multigroup hybrid model (MGHM) was designed with random forest for priority calculations and a support vector machine (SVM) for final classification.

For the baseline selection, as each group contained several descriptors, each group was compared by its best performance after feature selection. Separate random forest models were trained on each group; the importance of each feature was based on the GINI index [42], meaning that the information gain it could provide for each involved splitting. Then, in each group, an optimal subset that had the highest performance by AUC was found via SVM, while, naturally, the left-over variables built the complimentary set. $D_{i}^{0}$ was used to denote the baseline set and $D_{i}^{1}$ to denote the left-over set for group $D_{i}$. The optimal set that had the highest AUC was selected as the initial baseline; then, the proposed multi-level feature integration was performed on the rest of the groups. The integration sequence was in a descending order of the pre-evaluated AUC on the whole group level. This ranked set of descriptor groups was hereafter referred to as the descriptor pool (DP).

**Figure 4:**
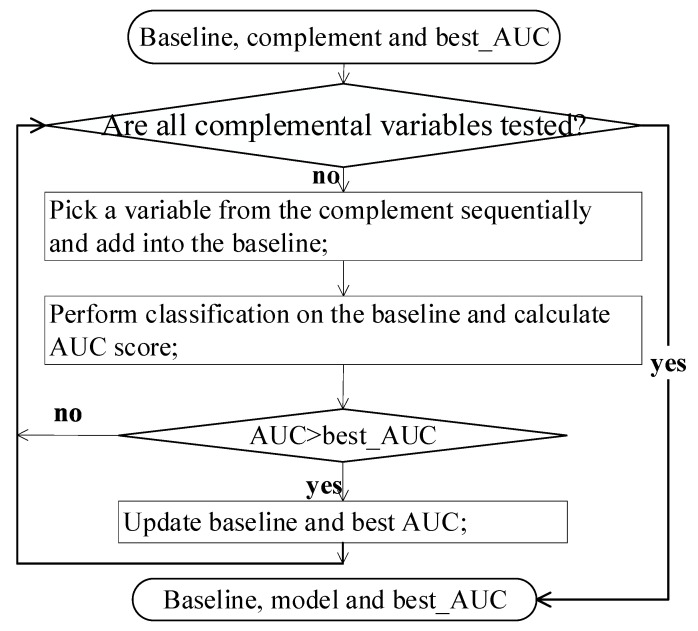
The flowchart of the feature selection step for the baseline and the complement in the multi-layer learning model.

Since there were three descriptor groups, the proposed hierarchical feature integration contained 4 levels. FSFS was performed on each level to find the optimal feature subset as output with support vector machine (SVM) as the classifier and the AUC as the metric, for which cross-validation evaluation was performed. Level *i* in the hierarchy model is denoted as $L_{i}$, the current baseline is denoted as $Baseline_{i}$, and the next candidate descriptor group in $L_{i}$ is denoted as $Candidate_{i}$. The output of $L_{i}$, denoted as $Baseline_{i+1}$, served as the baseline of $L_{i+1}$. Its flow chart is plotted in Figure 4. After all candidate sets were integrated, FSFS was run to integrate the complementary set of the initial baseline.

As this method was designed to iteratively evaluate every variable, it served as the upper-bound of the performance that can be achieved on the dataset.

**Figure 5:**
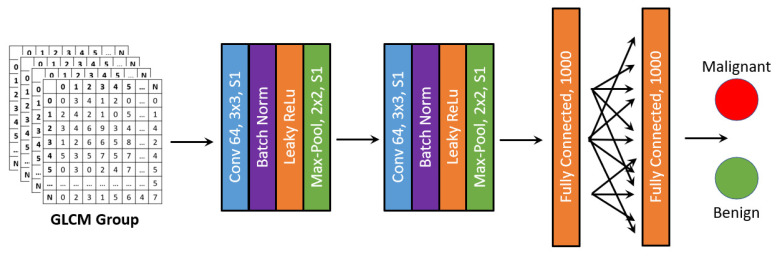
Network structure of FSFS-CNN.

**Multi-group CNN:** In the second model, CNN was adapted and performed adaptive learning by each group, as shown in Figure 5. For the baseline selection, the CNN was designed to take the whole GLCM group as input and select the one with the highest AUC. Then, the integration was performed by iteratively adding a group with the next highest AUC following FSFS. The entire evaluation was based on a CNN network, where its detailed structure is listed in Table 2 and the structure of the backbone is plotted in Figure 5. For each level, the input size of the network had 32 × 32 × c, where 32 is the grayscale and c is the number of channels/GLCMs of the input. The convolution network contained two convolution layers, each followed by a batch normalization layer, a max-pooling layer with stride 2 and ReLU as activation function. After the convolution part, three fully connected layers were designed to make a final prediction. For different group combinations, the number of input channels were modified to fit the current input data. This multi-group CNN method is denoted as MG-CNN in the rest of the paper.

## 3. Results

In this section, the polyp mass dataset used for all experimental results is discussed in detail. The classification results of the multi-scale descriptor sets are presented with the proposed multi-level adaptive learning model. Finally, the proposed models are compared to similar classification methods which input all the multi-scale descriptor sets at once and ignore the differences among the data sets.

### 3.1. Polyp Dataset

**Figure 6:**
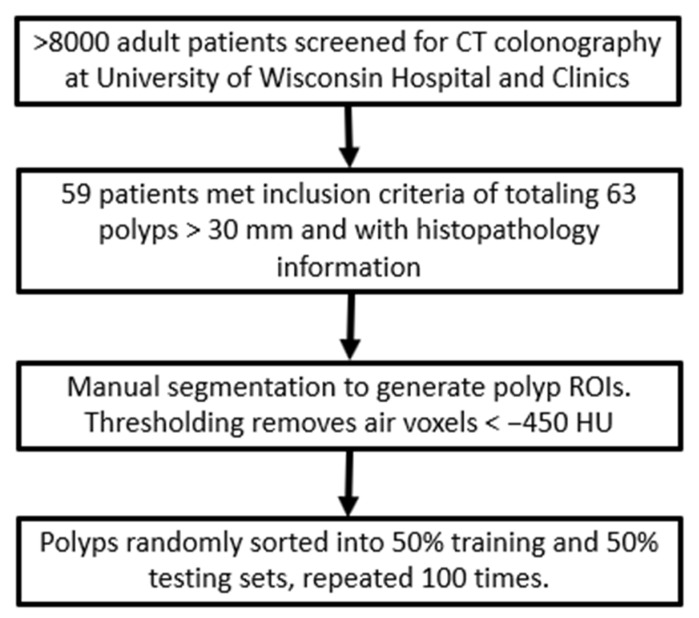
Flowchart of data acquisition and preparation for these experiments.

The polyp dataset used for these experiments consisted of 59 patients with a total number of 63 polyp masses found through virtual colonoscopy and confirmed by clinical colonoscopy. A flowchart of the dataset acquisition and preparation is shown in Figure 6 and described below. The polyp dataset used for these experiments was obtained from a retrospective study carried out at the University of Wisconsin Hospital and Clinics, Madison, WI, USA. Over 8000 patients were screened via CTC with the inclusion criteria that the patients were at least 50 years of age (normal screening age without family history of colorectal cancer), a polyp with a size of at least 30 mm in largest diameter was detected during CTC, and corresponding histopathology was available for those polyps. The CTC imaging was carried out according to the procedures described within [43]. Of those screened patients, only 59 patients, with a total of 63 polyp masses, fit the inclusion criteria. For classification discussed below, the dataset was divided into binary categories of 32 malignant adenocarcinomas, and 31 benign polyps including 3 serrated adenomas, 2 tubular adenomas, 21 tubulovillous adenomas, and 5 villous adenomas. All polyps had bulky mass morphology, except for six (four tubulovillous and two villous adenomas), which were designated as flat or carpet polyps. The patient demographics for this polyp dataset are presented in Table 3.

The clinical value of CADx models on CTC polyp mass images is due to their requirement for surgical removal from their size. Unlike endoscopic colonoscopy, CTC is noninvasive and cannot resect polyps during the procedure. Polyp masses that are 30 mm or larger in size, however, require surgical removal and are not treated via colonoscopy. Therefore, the clinical value of examining this dataset is to provide physicians with diagnostic information on the polyp masses before their surgical removal without requiring expensive biopsy procedures. For example, surgeons may decide to be more aggressive in how much tissue they remove if the mass is malignant to ensure that any microscopic disease which may have invaded surrounding tissues can also be removed.

#### 3.1.1. Regions of Interest

**Figure 7:**
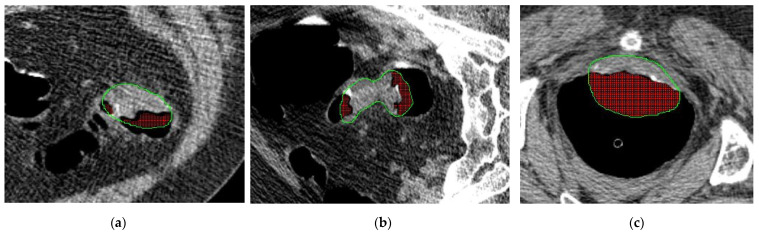
Three sample CT slices from select polyp masses. Green contour around the polyp show the segmentation. Air voxels from the lumen below −450 HU are removed post-segmentation and are highlighted red in the images. Images show sample polyps with pathologies (**a**) adenocarcinoma, (**b**) villous adenoma, and (**c**) villous adenoma.

The area around the polyp region was manually selected and segmented on each CTC image slice containing the polyp. For each polyp, a volume was constructed by combining the segmentations on each slice to form the region of interest (ROI), which was confirmed by radiologists to ensure accuracy of the manual procedure. It is noted that a cleansing step was used to discard all voxels below −450 HU within these ROIs as being predominately air from the lumen of the colon [44]. The information encoded in these voxels from partial volume effects (above the range of pure air HU values) is minimal, if any, and contributes more noise to the features for classification. The ROIs were used to compute the multi-scale texture features described above. Sample polyp CT slices and their contours are shown in Figure 7.

#### 3.1.2. Dataset Evaluation

A cross-validation strategy was used to evaluate the model performance. The leave-one-out and two-fold methods were adopted in this study to provide the two bounds of the classification performance, where the two evaluation methods were two extremes of the k-fold cross validation. The leave-one-out method tests only on one subject but trains on all the other subjects. The two-fold method trains on half the subjects and tests on the other half, which trains the model with the least data samples. This strategy is particularly attractive for small sized datasets. Results from both methods together will provide a fairer evaluation to consider the overfitting that might happen in the leave-one-out method and the lower amount of training that might happen in the two-fold method. Due to the paper length limit, only the two-fold testing results are used to show the advantage of the proposed model under the toughest conditions. The polyps were randomly divided into training and testing sets for classification with 31 polyps in the training set (15 benign and 16 malignant) and 32 polyps in the testing set (16 benign and 16 malignant). Repeating this random sampling method, 100 training and testing groups were generated to increase statistical confidence and to minimize bias. The 100 classification outcomes were averaged for the results and standard deviation (STD) served as the performance variation measurement.

#### 3.1.3. Settings

For the traditional method, three multi-scale descriptors were calculated using the three groups in Table 1 relevant to the three scales. Then, these descriptors were used to generate 100 training and testing datasets due to the observation splitting schemes.

The Random Forest classifier contains 5000 trees with GINI index as the importance metric. The SVM classifier adapts a kernel function of cubic polynomial, with Gamma as 1/(variable number), coef0 as 0, tolerance as 0.001, and Epsilon as 0.1.

For each learning method, the *i*-th candidate group is denoted as $D_{i}^{x}$, where iϵ[1,3] and xϵ[·,b,c] as the whole group, base group and complementary group. $C_{i}$, with iϵ[1,3], denotes the learned best set from stage *i*.

The CNN model is trained with Cross-entropy loss between the predicted score and label. Adam [45] was used for optimization. The learning rate was initialized as 0.001 and decayed by 0.01 every 10 epochs. Since the dataset was relatively small, the training ended after 40 epochs to prevent overfitting of the model.

### 3.2. The Outcomes of the Proposed Method

First, an investigation of how the descriptors from each group contribute to the model trained from all descriptors is analyzed. The statistics summary of the descriptors is listed in Table 1.

**Figure 8:**
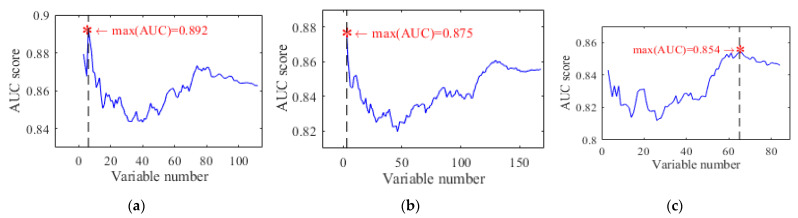
The trends of three AUC score curves of polyp classification, their maximums and their partitions over 63 polyps via forward step feature selection method: (**a**) D1, (**b**) D2, and (**c**) D3.

After acquiring the optimal subset of descriptors, the contribution of each group is analyzed by comparing how many variables contribute to the best AUC score and the importance of each descriptor. Figure 8 shows the different trends of AUC scores as a function of variable number, where the non-monotonic trend is usually seen due to the redundancy, resulting in parameter overtraining and clustering degradation. In addition, the differences among the multi-scale texture descriptors are also clearly seen.

Based on the observation above, it is necessary to evaluate each descriptor group first before combining them all together in order to avoid deterioration on the overall performance. Besides, this can prove the feasibility of the proposed learning framework.

The performance of the three groups of descriptors using the hybrid model were analyzed first. Among all, as shown in Table 4, the highest AUC is achieved by $D_{3}$ where 6 variables were chosen for this preliminary classification result. Following the proposed method, every ranked descriptor was divided into two parts, baseline and complementary set. The six generated subgroups, or the baseline and the compliment for each of the three descriptor groups, are shown in Table 5.

After the first step, based on AUC scores, DP was initialized as {$D_{3}^{b}$, $D_{3}^{c}$, $D_{2}^{b}$, $D_{2}^{c}$, $D_{1}^{b}$, $D_{1}^{c}$}. After selecting $D_{3}^{b}$ as the initial baseline, DP became {$D_{3}^{b}$, $D_{2}$, $D_{1}$, $D_{3}^{c}$}. Then, DP was fed into MGHL to remove the redundant variables and to improve classification performance via the proposed bottom-up hierarchical integration. Finally, 17 out of 364 variables were extracted to form the final descriptor. In terms of classification results, the AUC score increased from 0.892 to 0.925, while its standard deviation dropped from 0.098 to 0.035. The changes in AUC score and the chosen variables are listed in Table 6, which illustrates that the hybrid model has a monotonic learning process.

The preliminary classification performances of the MG-CNN are also listed in Table 4. When compared to the results of using the whole 13 directions, the results indicated that multiple directions of GLCM could contribute to the classification performance, which means that GLCM with different directions could provide additional information.

Then, $G_{1}$ with 3 GLCMs was chosen as the baseline, with the remaining two groups to be iteratively tested for whether they should be included. Finally, three subgroups were selected and contributed to a final 0.909 AUC score. In addition, classification performance from two-scales already achieved better classification performance than using all the directions without the multiscale concept. The hierarchical learning process is shown in Table 7 and illustrates that the feature integration scheme was indeed useful to further optimize the classification performance.

### 3.3. Comparisons with State-of-the-Art Models

In addition to the above presentation of the performance details of the adaptive learning model for integration of multiscale texture features, the comparisons to several typical state-of-the-art models are also detailed, including:

- Extended Haralick Measures (eHM)—this descriptor includes all the 364 variables derived from the 28 HMs over the 13 directions and disregards the multi-scale nature [11];
- Post-KL Transformation (KLT) eHMs (eHM+KLT)—this method combines eHMs and KLT to address the variation problem due to the multi-scale nature by the KLT [11];
- The Least Absolute Shrinkage and Selection Operator (LASSO)—a typical method of feature selection containing two steps, i.e., feature regularization and feature selection, for consideration of variation among feature datasets [28,34];
- The SVM Method with Recursive Feature Elimination (SVM-RFE)—another typical method in feature selection, including feature ranking and feature selection for consideration of variation among feature datasets [37];
- The Dependence Guided Unsupervised Feature Selection (DGUFS)—a new feature selection method applies the interdependence among original data, features, and labels in a joint learning framework to pick features [28];
- VGG16—a typical deep learning method, which is fed by 20 salient slices extracted from every polyp, where the feature extraction and selection operations are considered as learning processes [40];
- GLCM-CNN—the state-of-the-art of texture based deep learning model on the task of polyp diagnosis. It takes the whole 13-directional GLCM as input, ignoring the correlations among different groups to make decisions [40]. The network structure is optimized to fit the polyp dataset used.

**Figure 9:**
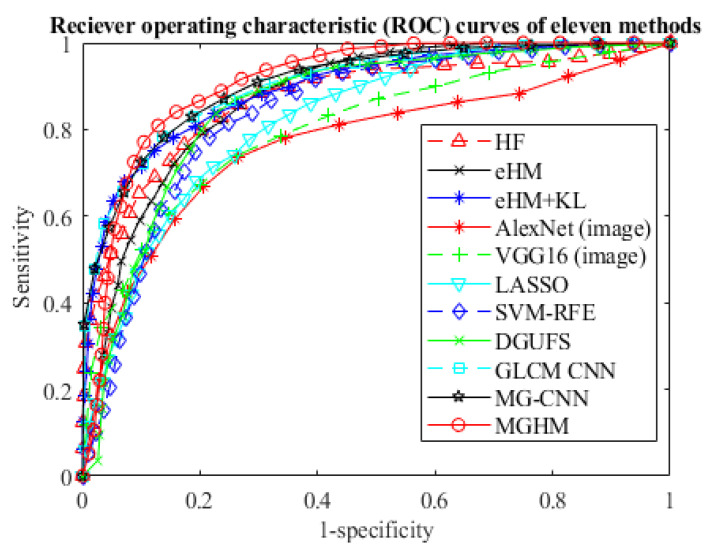
ROC curves of proposed and comparative methods.

Table 8 lists the classification performance of all the methods on the polyp mass dataset, where the AUC, accuracy, sensitivity, and specificity of each model is reported. The AUC score and accuracy of the proposed method exceeds that of the post-KLT eHMs (the best result of the six typical methods) by 2% and 3%, respectively. Against VGG-16, the proposed model improves the AUC score by 10%. Moreover, all ROC curves are also plotted in Figure 9, where the proposed model’s ROC curve is the top one among the seven. These ROC curves further demonstrate the advantage of the proposed method over the others. Based on the graphical judgement in Figure 9 and the quantitative measurements in Table 8, both results demonstrate the advantages of the two adaptive learning models over the rest of the methods by a large margin. Moreover, a significance test was performed, as shown in Table 9, by comparing their prediction probabilities with eight state-of-the-art methods. All the *p*-values are less than 0.05, which indicates that the proposed methods have significant differences from the comparative methods.

## 4. Discussion

In this paper, a multi-layer adaptive learning model architecture is proposed. Instead of simply concatenating all the multi-scale texture features together for classification, the proposed architecture not only integrates multi-scale texture descriptors in an adaptive manner to consider the associated variation among multiple datasets, but also provides an effective solution for information redundancy. The primary novelty of this proposed work was in the weighted grouping of the texture patterns and assigning greater contributions to those higher weighted groups, instead of using all features entered into the classifier at the same time. Two schemes, i.e., traditional machine learning-based and CNN-based, were designed to demonstrate this idea. The proposed design contained two stages. In the first stage, GLCM was divided into three groups by their individual scales. A baseline was selected, with the remaining groups ordered by their individual performance. In the second stage, the three group were integrated into one enhanced descriptor in a hierarchical architecture by a multi-layer learning scheme. On each layer, a forward stepwise feature selection method was introduced to selectively add some patterns or variables from complemental subgroups into the baseline to produce better performances. The greedy procedure guarantees a monotonically increasing AUC score from the initial descriptor groups at the first layer and reduces redundant information. Due to the variation among multiple datasets or multiscale descriptors, the proposed adaptive learning model increased the AUC score from 0.886 to 0.925 via MGHM and from 0.895 to 0.909 via MG-CNN.

When comparing against the deep learning state-of-the-art methods, the following observations were noted. The VGG16 and AlexNet models performed quite poorly, with AUC values of 0.823 and 0.779, respectively. These results were expected because deep learning methods tend to have much higher data requirements to fully train the high-level features from that methodology, and the dataset used for these experiments is relatively small. However, the proposed MG-CNN model still attained a significantly higher AUC value of 0.909. This showed that the GLCM input for the model already provided some higher-level texture information, so that the deep learning architecture did not have the same steep data requirements as the other methods. On a much larger dataset, it is expected that the VGG16 and AlexNet models will provide closer comparisons to the proposed models. Against the GLCM-CNN method, which was originally used on the same dataset as these experiments [40], the value of the proposed weighted grouping was demonstrated by the higher AUC value. Since the GLCM-CNN model similarly outperformed the VGG16 and AlexNet models, this further reinforced the value of the GLCM as inputs.

When comparing against the other state-of-the-art methods using traditional features and classifiers, the proposed MGHM still outperforms them significantly. In this category, the post-KLT eHMs obtained the best classification performance of the comparative methods likely because the KL transform provides a measure of reducing redundancy of the texture features through the change of basis representation. Against the other traditional feature selection methods, the value of the proposed model in further reducing variation and redundancy to achieve greater classification is even more significantly demonstrated by AUC values.

Although the presented adaptive learning model is implemented for integration of multiscale texture features, the integration strategy can be applied to fuse multimodal datasets, such as the polyp intensity images, the first derivative gradient image and the second order curvature images that were investigated in Song et al. [6] and Hu et al. [11]. While this work investigated spatial variations through the GLCM, this method may help expand upon those other models that integrated multiple feature sets. Future studies will look to expand on the multi-scale texture descriptors to include other types of descriptors and patterns into a study with a larger dataset.

## Figures and Tables

**Table 1:** Digital direction subdivision by their voxel distances from one voxel to the concerned center voxel.

	Radius = 1	$\mathrm{Radius}=\sqrt{2}$	$\mathrm{Radius}=\sqrt{3}$
Direction group ID	$G_{1}$	$G_{2}$	$G_{3}$
Number of GLCM Directions	3	6	4
Descriptor group ID	$D_{1}$	$D_{2}$	$D_{3}$
Number of variables	84	168	112

**Table 2:** Detailed network design for MG-CNN.

Structure	Type	Kernel Size	# of Kernels/ Channels/Neurons/Strides	Activation
Layer 1	2D Convolution	3 × 3	64 (stride 1)	ReLU
Layer 2	Batch Normalization		64	
Layer 3	Maxpool	2 × 2	(stride 2)	
Layer 4	2D Convolution	3 × 3	64 (stride 1)	ReLU
Layer 5	Batch Normalization		64	
Layer 6	Maxpool	2 × 2	(stride 2)	
Layer 7	Dense		1000	ReLU
Layer 8	Dense		1000	ReLU
Layer 10	Dense		2	softmax

**Table 3:** Patient demographics of polyp mass dataset.

Pathology	Count	Class	Male: Female	Average Age (yrs)	Average Size (mm)
Tubular Adenoma	2	0	2:0	69.8	35.0
Serrated Adenoma	3	0	2:1	55.2	34.3
Tubulovillous Adenoma	21	0	11:10	64.4	36.9
Villous Adenoma	5	0	4:1	67.4	55
Adenocarcinoma	32	1	12:20	69.9	43.9

**Table 4:** The preliminary classification results of the two proposed models.

Group ID	GLCM Directions	MGHM AUC (Mean ± STD)	MG-CNN AUC (Mean ± STD)
$G_{1}$	3	0.846 ± 0.098	0.895 ± 0.064
$G_{2}$	6	0.875 ± 0.101	0.889 ± 0.061
$G_{3}$	4	0.892 ± 0.098	0.871 ± 0.074

**Table 5:** Two parts of each descriptor divided by forward step feature selection method via SVM classifier.

Descriptor-ID	$D_{1}$		$D_{2}$		$D_{3}$	
AUC Score	0.854		0.875		0.892	
Sub-ID	$D_{1}^{b}$	$D_{1}^{c}$	$D_{2}^{b}$	$D_{2}^{c}$	$D_{3}^{b}$	$D_{3}^{c}$
Number of Variables	65	19	3	165	6	106

**Table 6:** All results of the MGHM over the polyp dataset. Descriptor pool represents the current candidates and its sequence in each layer. $Baseline_{2}$ and $Baseline_{3}$ are two new descriptors generated by the baselines and the complements of their previous layers.

Layer	Baseline			Candidate		Descriptor Pool (DP)
	Source	Variables	AUC (Mean ± STD)	Source	Selected Variables	
$L_{1}$	$Baseline_{1}\;\left(D_{3}^{b}\right)$	6	0.892 ± 0.098	$D_{2}$	4	$D_{2},\;D_{1},\;D_{3}^{c}$
$L_{2}$	$Baseline_{2}$	10	0.916 ± 0.038	$D_{1}$	3	$D_{1},\;D_{3}^{c}$
$L_{3}$	$Baseline_{3}$	13	0.919 ± 0.036	$D_{3}^{c}$	4	$D_{3}^{c}$
$L_{4}$	Final Descriptor	17	0.925 ± 0.035			-

**Table 7:** The results of MG-CNN over the polyp dataset. Descriptor pool represents the current candidates and its sequence in each layer. $Baseline_{2}$ is a new descriptor generated by the baselines and the complements of their previous layers.

Layer	Baseline			Candidate		Descriptor Pool (DP)
	Source	GLCMs	AUC (Mean ± STD)	Source	GLCMs	
$L_{1}$	$Baseline_{1}\;\left(G_{1}\right)$	3	0.895 ± 0.064	$G_{2}$	4	$G_{2},\;G_{3}$
$L_{2}$	$Baseline_{2}$	9	0.904 ± 0.047	$G_{3}$	3	$G_{3}\;$
$L_{3}$	Final Results	13	0.909 ± 0.051			

**Table 8:** Four evaluation measurements of proposed and comparative methods.

Method	AUC	Accuracy	Sensitivity	Specificity
eHM	0.886	0.797	0.868	0.726
eHM+KLT	0.907	0.814	0.781	0.848
AlexNet (image)	0.779	0.778	0.831	0.726
VGG16 (image)	0.823	0.741	0.714	0.769
LASSO	0.836	0.748	0.791	0.706
SVM-RFE	0.856	0.783	0.775	0.791
DGUFS	0.866	0.806	0.836	0.776
GLCM CNN	0.900	0.856	0.843	0.868
MG-CNN	0.909	0.864	0.866	0.862
MGHM	0.925	0.884	0.891	0.878

**Table 9:** *p*-values from statistical significance analysis over the ten methods using Wilcoxon Signed-rank Test between the predicted probabilities of these methods.

Method	eHM	eHM+KLT	AlexNet	VGG16	LASSO	SVM-RFE	DGUFS	GLCM CNN
MGHM	<<0.05	0.0179	<<0.05	<<0.05	<<0.05	<<0.05	<<0.05	0.0204
MG-CNN	<<0.05	0.0411	<<0.05	<<0.05	<<0.05	<<0.05	<<0.05	0.0398

## Data Availability

All data used for these experiments can be made available from the contact author upon reasonable request.
